# Supplementary material for: Content-rich biological network constructed by mining PubMed abstracts
Source: BMC Bioinformatics. 2004 Oct 8;5:147. doi: 10.1186/1471-2105-5-147 (PMC528731; doi:10.1186/1471-2105-5-147)
Supplement: Additional File 2 — The original results of the above study (non-essential files are deleted to keep the file size under the limit set by BMC bioinformatics). [file 1471-2105-5-147-S2.bz2 › chilibotAdditionalFile2/dip20/515ID8799122E10818/html/ESR1_CREBBP.html]

 


 **ESR1** and **CREBBP** 
  
Found 57 abstracts in PubMed, retrieved 20.  
 

 What does Google say? 
 PDF only 
| .edu only 

---

**Interactive relationship** (e.g. stimulation, inhibition, etc)

**Inhibitory relationship**- These findings suggest that the cofactors p300 and CBP  [ **CREBBP** ]  modulate the ability of the BRCA1 protein to inhibit ER alpha  [ **ESR1** ]  signaling.  Ref: 11782371 Cancer Res, 2002
- These results suggest that 17beta estradiol bound estrogen receptor  [ **ESR1** ]  may inhibit nuclear factor kappaB dependent transcription of RANTES gene by competing with p65 for limiting amounts of CREB binding protein  [ **CREBBP** ] .  Ref: 12603855 J Invest Dermatol, 2003
**Stimulatory relationship**- Regulation of GRIP1 and CBP  [ **CREBBP** ]  Coactivator activity by Rho GDI modulates estrogen receptor  [ **ESR1** ]  transcriptional enhancement.  Ref: 12138084 J Biol Chem, 2002
- In addition, we show that expression of p21 WAF1 CIP1 SDI1 alleviates the block on CBP  [ **CREBBP** ]  function mediated by Cdk2 and in turn stimulates transcriptional activation by ER alpha  [ **ESR1** ]  in a CBP  [ **CREBBP** ]  histone acetyltransferase HAT dependent manner.  Ref: 12173048 Oncogene, 2002
- Further, 4EM mediated transcription in ER alpha  [ **ESR1** ] , like estrogen, was enhanced in the presence of coactivators, steroid receptor coactivator 1 SRC 1, CREB binding  [ **CREBBP** ]  proteins CBP  [ **CREBBP** ] , and E6 associated protein E6 AP.  Ref: 12732288 J Steroid Biochem Mol Biol, 2003
**Neutral relationship**- RTS  [ **CREBBP** ]  offers new promising possibilities in the postgenomic era  [ **ESR1** ] .  Ref: 12570786 Curr Protein Pept Sci, 2003
- Ligand Independent Interactions of p160 SRC Coactivators and CBP  [ **CREBBP** ]  with Estrogen Receptor alpha  [ **ESR1** ]  Regulation by Phosphorylation Sites in the A B Region Depends on Other Receptor Domains.  Ref: 12714702 Mol Endocrinol< MedlineTA>Mol Endocrinol, 2003
- Mediator and p300 CBP  [ **CREBBP** ]  steroid receptor coactivator complexes have distinct roles, but function synergistically, during estrogen receptor alpha  [ **ESR1** ]  dependent transcription with chromatin templates.  Ref: 12482985 Mol Cell Biol, 2003
- The rescue function mapped to the cysteine histidine rich domain CH3, a region of p300 CBP  [ **CREBBP** ]  that we found to interact directly with the conserved COOH terminal activation domain AF 2 of ER alpha  [ **ESR1** ] .  Ref: 11782371 Cancer Res, 2002

**Non-interactive relationship** (e.g. studied together, co-existance, homology, etc.)

- We have used a biochemical approach, including an in vitro chromatin assembly and transcription system, to examine the functional role for Mediator in the transcriptional activity of estrogen receptor alpha  [ **ESR1** ]  ERalpha with chromatin templates, as well as functional interplay between Mediator and p300 CBP  [ **CREBBP** ]  during ERalpha dependent transcription.  Ref: 12482985 Mol Cell Biol, 2003
- ... two other nuclear receptor coactivators, the p300 CBP  [ **CREBBP** ]  associated factor PCAF and the glucocorticoid receptor interacting protein 1 GRIP1, failed to rescue the ER alpha  [ **ESR1** ]  activity.  Ref: 11782371 Cancer Res, 2002
- Furthermore, not only AR but also the glucocorticoid receptor YFP, ER alpha  [ **ESR1** ]  GFP, and YFP tagged SRC 1, TIF2, and CBP  [ **CREBBP** ]  were found to be accumulated in identical spots in the presence of ligand.  Ref: 11923466 Mol Endocrinol, 2002
- In addition, we examined the preference of E2 bound ER alpha  [ **ESR1** ]  and ER beta for various naturally occurring NR boxes including the 10 SRC boxes as well as the motifs from PGC 1, TRBP, TRAP220, and CBP  [ **CREBBP** ] .  Ref: 12126937 Mol Genet Metab, 2002
- Further experiments demonstrated that overexpression of the transcriptional coactivator cAMP response element binding protein CREB binding protein  [ **CREBBP** ]  CBP  [ **CREBBP** ]  p300, a factor common to both the estrogen receptor  [ **ESR1** ]  and HIF pathways, eliminated E2beta mediated attenuation of hypoxia induced ET 1 promoter activity.  Ref: 12060564 Am J Physiol Lung Cell Mol Physiol, 2002
- These results suggest a novel mechanism by which p21 WAF1 CIP1 SDI1 functions as an enhancer of ER alpha  [ **ESR1** ]  activity through the modulation of CBP  [ **CREBBP** ]  function.  Ref: 12173048 Oncogene, 2002
